# From Bacterial Toxin to Therapeutic Agent: The Unexpected Fate of Mycolactone

**DOI:** 10.3390/toxins15060369

**Published:** 2023-05-30

**Authors:** Daniela Ricci, Caroline Demangel

**Affiliations:** Institut Pasteur, Université Paris Cité, Inserm U1224, Immunobiology and Therapy Unit, 75015 Paris, France; daniela.ricci@pasteur.fr

**Keywords:** mycolactone, *Mycobacterium ulcerans*, Sec61, immunomodulation, viral infection, cancer

## Abstract

“Recognizing a surprising fact is the first step towards discovery.” This famous quote from Louis Pasteur is particularly appropriate to describe what led us to study mycolactone, a lipid toxin produced by the human pathogen *Mycobacterium ulcerans*. *M. ulcerans* is the causative agent of Buruli ulcer, a neglected tropical disease manifesting as chronic, necrotic skin lesions with a “surprising” lack of inflammation and pain. Decades after its first description, mycolactone has become much more than a mycobacterial toxin. This uniquely potent inhibitor of the mammalian translocon (Sec61) helped reveal the central importance of Sec61 activity for immune cell functions, the spread of viral particles and, unexpectedly, the viability of certain cancer cells. We report in this review the main discoveries that marked our research into mycolactone, and the medical perspectives they opened up. The story of mycolactone is not over and the applications of Sec61 inhibition may go well beyond immunomodulation, viral infections, and oncology.

## 1. Introduction

Buruli ulcer (BU) is a neglected tropical disease caused by skin infection with the environmental pathogen *Mycobacterium ulcerans*. Following inoculation into subcutaneous tissues, the bacteria establish a local infection that escapes the control of the immune system and causes chronic ulcers characterized by a relative lack of inflammation and pain [1]. In 1999, pioneering studies conducted by George et al. revealed that *M. ulcerans* contains a plasmid coding for mycolactone, a polyketide synthase product underpinning bacterial pathogenicity and virulence [2,3]. Indeed, bacteria genetically modified to be mycolactone-deficient were unable to survive in experimentally infected guinea pigs, and the subcutaneous injection of purified mycolactone in these animal models was sufficient to induce BU-like lesions [2,3]. Why significant amounts of bacteria and dead cells in Buruli ulcers do not trigger inflammatory responses and pain, and how mycolactone contributes to these abnormalities, were intriguing questions that we decided to investigate in the laboratory [3].

## 2. The Immunomodulatory Effects of Mycolactone

We and other research groups started to investigate the dual, immunomodulatory and cytotoxic effects of mycolactone in models of immune cells, using mycolactone purified from bacterial cultures with a protocol adapted from the initial methodology developed by George et al. [2,4]. From these cellular studies, mycolactone was found to be a double-trigger weapon of *M. ulcerans* capable of rapidly paralyzing the effector functions of immune cells in conditions not affecting their viability, while inducing their apoptosis in the longer term [3]. Notably, mycolactone impacted both the innate and adaptive components of immunity, and in multiple ways [1]. In particular, it suppressed the ability of multiple immune cells (macrophages, dendritic cells (DCs), neutrophils, lymphocytes) to produce cytokines and chemokines upon activation [5,6,7,8,9]. Intriguingly, mycolactone did not affect the production of cytokines and chemokines at the mRNA level, and it did not affect protein synthesis in a global way. Cytokine production was interrupted at the intracellular level, pointing to a defect upstream of the secretion pathway.

Cytokines and chemokines were not the only proteins affected by mycolactone, and membrane receptors whose expression by immune cells was impacted by mycolactone were subsequently discovered. A striking example is L-selectin (also called CD62-L), a membrane protein that is expressed at high levels by naive T lymphocytes and mediates their trafficking to the draining lymph nodes through molecular interaction with high endothelial venules [10]. We found that mycolactone treatment downregulates the basal expression of CD62-L by human T cells in vitro, thereby altering their capacity to traffic to peripheral lymph nodes in vivo. Other examples of membrane proteins that were found highly susceptible to mycolactone treatment include cytokine receptors, such as the interferon (IFN) receptors. In T lymphocytes exposed to mycolactone in vitro, we observed within 6 h a significant decrease in cell surface expression of the receptors of IFN-α and IFN-γ. Similarly in macrophages, mycolactone rapidly decreased the cell surface expression of the IFN-γ receptor, reducing their ability to express nitric oxide synthase upon stimulation with IFN-γ, and thereby their antimicrobial functions [11].

Given their central role in initiating adaptive immune responses and immunity to mycobacteria [12], we were particularly interested to characterize the effects of mycolactone on the viability and functional biology of DCs. Both the phenotypic and functional maturations of DCs were inhibited by treatment with non-cytotoxic concentrations of mycolactone, leading to a defective emigration capacity of DCs upon adoptive transfer in mice, a decreased ability to activate allogeneic T cell priming and to produce inflammatory cytokines and chemokines upon in vitro stimulation [5]. A unique feature of DCs is their ability to capture antigens released by the surrounding cells and present them in the context of major histocompatibility complex class I (MHC-I) molecules, a process referred to as cross-presentation. In collaboration with the group of Sebastian Amigorena (Institut Curie, Paris, France), we showed that mycolactone dramatically affects DC production of MHC-I molecules, and therefore their ability to cross-present antigens [13].

Collectively, these data revealed mycolactone as a novel type of natural immunosuppressor, with the ability to prevent the generation of anti-mycobacterial responses at multiple stages [3]. Because mycolactone shared structural and functional features with the macrocyclic triene rapamycin and the macrolide lactone FK506, both immunosuppressive compounds operating through interaction with the intracellular receptor FKBP12 to inhibit the mammalian target of rapamycin C1 (mTORC1), we hypothesized that it could target the same signaling pathway [5]. While an inhibitory effect of mycolactone on mTOR signaling was reported by Bieri et al. [14], mycolactone did not alter the lipopolysaccharide-driven activation of mTORC1 in monocytes in an independent study by Simmonds et al. [6]. In our hands, mycolactone did not alter the constitutive activation of mTORC1 in the Jurkat T cell line, or the TCR-induced activation of mTORC1 in peripheral blood-derived lymphocytes [7]. Having excluded the known mechanisms of immune suppression and highlighted the unique immunosuppressive features of mycolactone, we concluded that mycolactone operates via a novel, selective and post-transcriptional mechanism of protein biogenesis inhibition.

## 3. Mycolactone Targets the Sec61 Translocon

With these discoveries, identifying the molecular target of mycolactone took a new dimension. Indeed, in addition to providing an effective treatment of BU, it could lead to the generation of novel immunomodulatory molecules. In 2014, Hall et al. made a breakthrough by demonstrating that mycolactone prevents the translocation of model secretory proteins into the endoplasmic reticulum (ER), leading to their degradation in the cytosol by the ubiquitin–proteasome system [15]. Using cell-free assays, McKenna et al. then showed that mycolactone selectively affects the step of cotranslational translocation of secreted and integral transmembrane proteins (TMPs) into the ER [16]. In eukaryotes, the cotranslational protein translocation pathway is initiated by recognition of signal peptides or transmembrane domains by the signal recognition particle (SRP). The SRP then targets the ribosome-nascent polypeptide complex to the Sec61 translocon. Sec61 is a heterotrimeric complex embedded in the ER membrane that ensures the transport of most secreted proteins (with a signal peptide but no transmembrane domain) and single-spanning TMPs into the ER. TMPs exclusively relying on Sec61 for insertion into the ER membrane include the Type I (with a signal peptide) and the Type II (without a signal peptide and a cytosolic N terminus) TMPs. Instead, the rare subsets of Type III TMPs (without a signal peptide and the opposite N terminal topology) and the C-terminal tail-anchored proteins can use alternative pathways for membrane integration at the ER [17,18], while mitochondrial membrane proteins depend on independent TIM/TOM complexes for mitochondrial membrane insertion.

In collaboration with Ville Paavilainen (University of Helsinki, Helsinki, Finland), we demonstrated that mycolactone prevents the cotranslational translocation of proteins into the ER by directly targeting the Sec61 translocon. Indeed, a single amino acid mutation (R66G) in the pore-forming (alpha) subunit of Sec61 conferred full resistance to mycolactone activity in bioassays [11]. Mycolactone was not the first reported inhibitor of Sec61. In 2005, Garrison et al. had discovered cotransin, a fungal product derivative with the capacity to inhibit Sec61 in a selective, substrate-specific manner [19,20]. Competition assays between cotransin and mycolactone, and mutant Sec61 studies, indicated that the binding sites of the two compounds overlap [11]. However, our in vitro translocation assays and global profiling of mycolactone-susceptible proteins in T cells, DCs and sensory neurons showed that, contrary to cotransin, mycolactone is not substrate-selective [11,13,21]. In addition, these proteomic analyses made it possible to characterize for the first time the signature of the Sec61 blockade at the cellular level [21].

Consistent with its mechanism of action, most of the detected Sec61 clients (secreted proteins, Type I and Type II TMPs) were massively downregulated by mycolactone in the three tested cell types. In contrast, mycolactone did not affect the cellular levels of Type III TMPs, C-tail anchored and mitochondrial membrane proteins [21]. Recent cryo-EM studies suggest that mycolactone and cotransin both operate by maintaining Sec61 in a close, inactive conformation through interactions with its sealing plug and lateral gate. However, the structural determinants of their differential selectivity for Sec61 substrates remain to be elucidated [22].

## 4. Structure–Activity Relationships: The Input of Synthetic Chemistry

**Figure 1:**
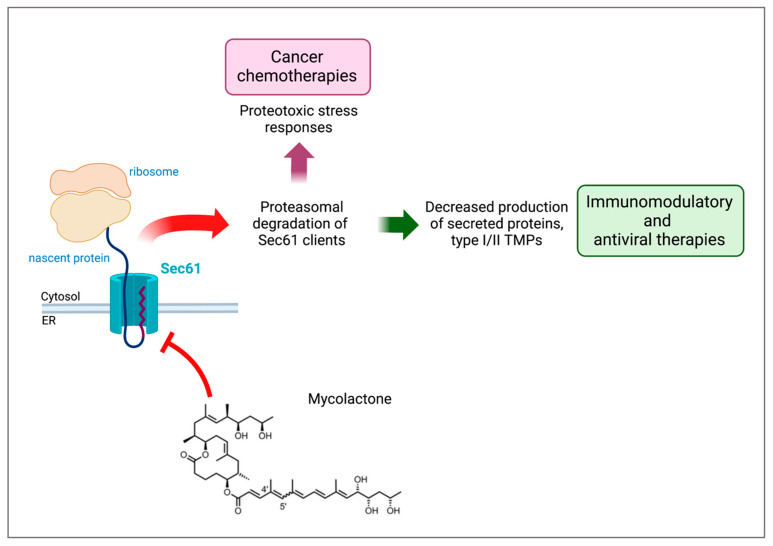
Sec61 blockade, its consequences at the cellular level and potential therapeutic applications. Mycolactone binds to Sec61 and blocks the translocation of its protein clients (secreted proteins and type I/II TMPs) in the ER, which in turn induces their degradation by the proteasome in the cytosol. This leads on the one hand to decreased levels of Sec61 clients, making it a potential immunomodulatory and antiviral agent, and on the other hand to proteotoxic stress responses and eventually to apoptosis, a useful addition to the cancer chemotherapies portfolio.

Identifying the minimal structural determinants of biological activity is key for the development of mycolactone-inspired derivatives that are compatible with large-scale synthesis. Since the initial description by George et al. [2], other structurally divergent mycolactones from genetically related mycobacterial species have been discovered and functionally characterized [23,24,25]. The most active in cellular assays is mycolactone A/B, produced by a subset of *M. ulcerans* clinical isolates (referred to as mycolactone in this review and depicted in Figure 1) [26]. The structure of mycolactone A/B is defined by a macrolactone ring with 12 atoms substituted at C11 by a C12–C20 carbon chain with hydroxyl functions at its end, and at C5 by a pentaenic chain C1′–C16′. In the natural state, mycolactone A/B is a mixture of geometric isomers bearing C4′–C5′ unsaturation with a Z-/E- ratio of 3:2. The Z-isomer defined as mycolactone A is in the majority due to the allylic constraints A of the E-isomer (mycolactone B).

Because *M. ulcerans* slowly multiplies and must be grown in safety laboratories of Level > 2, significant efforts were made to generate synthetic mycolactone and variants. In 2002, Song et al. reported the first total synthesis of mycolactone [27]. Additional synthetic routes engineered by different groups followed (Yin et al. [28], Feyen et al. [29] and Chany et al. [30]), which altogether provided researchers with a synthetic compound that was equivalent to the *M. ulcerans* factor in cellular assays. These stereo-selective, time-consuming syntheses involving at least 30 steps used different synthetical concepts, making a strong contribution to organic synthesis and paving the way for structure–activity relationship (SAR) studies [24].

One major finding of these SAR studies was that truncating mycolactone’s structure in any way is detrimental to its biological activity. This suggested that mycolactone is the result of an evolution of the pathogen for maximal inhibition of the host Sec61. In particular, an intact and full-length lower side chain of mycolactone was critical for Sec61 inhibition, and the core structure devoid of polyketide chains was biologically inert [31,32]. Using a diverse set of C8-desmethylmycolactone analogues generated by our collaborator Nicolas Blanchard (University of Haute Alsace, Mulhouse, France), we attempted to identify the simplest synthetic version of mycolactone presenting an optimal ratio between immunomodulatory and cytotoxic properties [8,30]. All synthetic compounds were compared to natural mycolactone for their capacity to block the activation-induced production of interleukin (IL)-2 by the human Jurkat T cell line (as a read-out of their immunomodulatory properties), and for their cytopathic activity in the human epithelial cell line HeLa (an epithelial cell model that is highly susceptible to mycolactone-mediated anoikis). A structural module of natural mycolactone lacking the upper side chain and core C8-methyl (hereafter named mini-mycolactone) was selected. In further assays using human primary cells, mini-mycolactone significantly retained natural mycolactone’s ability to inhibit the production of key inflammatory cytokines such as the tumor necrosis factor (TNF)-α, while displaying a relatively lower cytotoxicity in primary dermal fibroblasts modeling ulcerative activity [8]. Therefore, in addition to being synthetically simpler than the natural product, mini-mycolactone was expected to display a broader therapeutic window in vivo.

## 5. Sec61 Activity, Immunity and Apoptosis

The identification of mycolactone-resistant Sec61 mutants enabled us to examine the role of the translocon in its immunomodulatory and cytotoxic effects. Production of IFN-γ by T cells and activation of the IFN-γ receptor signaling pathway in infected macrophages are key components of anti-mycobacterial immunity [33]; both of which were markedly impaired by mycolactone. Expression of the Sec61α-R66G mutant in mycolactone-treated T cells rescued their effector functions in vitro and in vivo. When expressed in macrophages, the mycolactone-resistant mutant restored IFN-γ receptor-mediated anti-microbial responses [11]. These experiments demonstrated that Sec61 is the host receptor mediating the diverse immunomodulatory effects of mycolactone. Further, they revealed a novel mechanism of immune evasion evolved by *M. ulcerans*. Beyond the control of IFN-γ and IFN-γ receptor production, our proteomic studies showed that inhibiting protein translocation has the potential to suppress inflammatory responses. In mouse models, the systemic administration of mycolactone could be used therapeutically to limit chronic skin inflammation, rheumatoid arthritis and inflammatory pain [8] (Figure 1). While less potent than natural mycolactone in the conditions used, mini-mycolactone also conferred significant protection in these disease models, confirming the potential of mycolactone-derived structures as prospective immunosuppressants.

Notably, the Sec61α-R66G mutant protected mycolactone-treated cells from undergoing apoptosis [11], showing that mycolactone cytotoxicity is a late consequence of Sec61 blockade. Looking at the proteins that were up-regulated by mycolactone in treated cells, we identified the hallmarks of cytosolic and ER stress responses. Thapsigargin, tunicamycin, and MG132 are canonical ER stressors targeting Ca^2+^ ATPases, protein glycosylation or the proteasome, respectively, which trigger an unfolded protein response (UPR) to restore protein homeostasis. Like them, mycolactone upregulated the UPR in treated cells, leading to the expression of the pro-apoptotic factor C/EBP homologous protein (Chop). This provided an explanation for how a prolonged exposure to saturating amounts of mycolactone can lead to cell apoptosis (Figure 1). However, unlike canonical ER stressors, mycolactone did not augment in parallel the expression of the ER chaperone GRP78/BiP [21,34]. Because BiP increases the cell’s ability to resolve ER stress and prevents the transition from protective to terminal UPR, this suggested that ER stress caused by mycolactone is more prone to evolve towards apoptosis.

## 6. Sec61 Blockers for Oncology

Our observation that mycolactone causes terminal UPR led us to investigate whether the proteotoxic impact of Sec61 blockade could also be exploited therapeutically. Despite the fact that considerable advances have been made over the years, the plasma cell malignancy multiple myeloma (MM) still remains an incurable disease. The current first line of treatment consists of proteasome inhibitors (bortezomib and derivatives) and immunomodulators (lenalidomide and derivatives), but eventually the majority of MM patients will relapse over time because of the generation of drug-resistant cancerous cells [35]. Therefore, the development of novel drugs with different mechanisms of action is vital to turn the tide of the battle against MM. We reasoned that Sec61 blockade may represent a novel therapeutic approach of interest in MM, by inducing proteotoxic stress responses while preventing the expression of membrane receptors that are key to MM cell division and dissemination.

With regard to this, recent work in our laboratory established protein translocation inhibition as a novel, useful tool against MM. By broadly inhibiting Sec61 with mycolactone, we showed that the translocon’s activity has a central role in determining MM cells’ fate. Indeed, mycolactone efficiently reduced MM cell line production of immunoglobulins and multiple type I/II TMP receptors such as CD138, a hallmark of MM that allows the survival of cancerous cells in the bone marrow by promoting growth factor signaling. Mycolactone treatment also decreased MM cell expression of the pro-survival IL-6 receptor and CD40, whose activation stimulates IL-6 production. As a later effect, mycolactone induced a pro-apoptotic ER stress-response in MM cell lines and tumors isolated from MM patients. This was used as proof of concept to show that Sec61 inhibitors have the potential to be used as an anti-cancer treatment against MM, alone or in combination with currently used chemotherapies [36]. Strikingly, mycolactone combined with bortezomib significantly delayed MM tumor growth in mice without significant toxicity. Equally importantly, mycolactone showed a synergistic action with lenalidomide, and was even effective at inducing cell death in bortezomib- and or lenalinomide-resistant cells [36,37], giving hope for the treatment of relapsed/refractory MM (Figure 1).

As described in the previous sections of this review, type III TMPs translocate into the ER in a Sec61-independent manner, and their levels are therefore not blunted by Sec61 inhibitors. The plasma cell-specific B cell maturation antigen (BCMA) belongs to the class of type III TMPs and ever since its overexpression and activation have been associated with MM, it has attracted great attention from the medical research community. As a result, BCMA is currently used as a biomarker for MM diagnosis and tracking. Furthermore, BCMA is used as a target to treat MM through specific antibodies and chimeric antigen receptor (CAR)-T cell therapy, both of which are giving promising results in clinics for an efficient and durable MM cancer treatment [38]. As expected, we found that mycolactone does not decrease BCMA expression via MM cell lines, but surprisingly its expression was greatly increased after treatment in a dose-dependent manner [36]. Even though we do not have mechanistic insights as to why mycolactone increases BCMA levels, we hypothesize that this is a secondary effect of the stress response induced by the Sec61 blocker. Nonetheless, it stands to reason that the effect of mycolactone on BCMA may potentiate the anti-BCMA therapies that are currently emerging against MM, giving mycolactone an additional key role in MM treatment.

Mycolactone is not the only Sec61 inhibitor that showed promising results by targeting clinically relevant proteins in the Oncology field. In fact, the cyclodepsipeptide apratoxins, a class of molecules isolated from marine cyanobacteria, showed a significantly strong antiproliferative activity in different cancer cells and only later was its mechanism of action revealed. Like cotransin and mycolactone, apratoxin A inhibits the secretion pathway through the direct inhibition of Sec61 [39]. The ability of apratoxin A to inhibit vascular endothelial growth factor (VEGF)-A expression inspired Cai et al. to test its anti-angiogenic effect, a hallmark of solid tumors. The group showed that a synthetic analog of apratoxin A, which was improved through structure–activity relationship studies and named apratoxin S10, inhibits angiogenesis and cancer cell growth in vitro. The antiangiogenic effect was mainly due to the ability of apratoxin S10 to downregulate VEGF receptor on endothelial cells while the anti-tumor effect was shown to result from a reduction in secretion of VEGF-A and IL-6, which have a role in both promoting tumor cell growth and the formation of new blood vessels [40]. The dual anti-tumor and anti-angiogenic properties of apratoxin A are extremely promising for solid tumor treatments, but the extensive pancreatic toxicity of the molecule in vivo [39] has blocked it from further clinical studies. How it compares to mycolactone and cotransin, in terms of Sec61 substrate selectivity and mechanism of inhibition, remains largely unknown.

As mentioned above, a different molecule belonging to the same family, the cyclodepsipeptide natural product cotransin, which was first discovered through a Sec61-unrelated screen of molecules that aimed at discovering inhibitors of the expression of cell adhesion molecules, proved to be a selective Sec61 inhibitor. Unlike mycolactone, cotransin only inhibits a subset of Sec61 clients, including the vascular cell adhesion molecule 1 (VCAM-1) and human epidermal growth factor receptor 3 (HER3), by preventing the recognition of specific signal sequences at the N-terminus end of target proteins by the translocon [20]. HER3 is a therapeutically interesting Sec61 client because it has a key role in tumorigenesis in several types of tumors, induces cell proliferation and has been linked to chemotherapy resistance. Much effort has been devoted to targeting HER3 through diverse methods against different types of cancers [41]. Ruiz-Saenz et al. showed that CT8, which belongs to the class of cotransins, selectively inhibits the expression of HER3 by blocking its translocation in the ER and subsequently inducing its proteasomal degradation in the cytosol, with no effect on the expression of other protein members of the HER family. This selective effect was in fact due to the specific sequence of the N-terminal signal peptide of HER3, as part of the mechanism of action of cotransins, and proved to enhance the pro-apoptotic effects of chemotherapeutic drugs currently used against breast cancer and other solid tumors [42].

What we learned from the action of these natural products shed light on the great therapeutic potential of Sec61 blockers: their anti-tumor, pro-apoptotic properties, together with the possibility of developing specific inhibitors that target selective clinically relevant Sec61 proteins to avoid/minimize normal cell toxicity, show that Sec61 inhibitors may make great contributions to the field of Oncology in the near future.

## 7. Indications beyond Inflammation and Oncology

In recent years, the potential of Sec61 inhibitors has been explored as a weapon against other threats to global public health such as viral infections (Figure 1). Viruses take advantage of many cellular machineries to enter the cell and to replicate themselves and the translocon is one of those, which viruses use to form mature viral particles ready to perpetuate the infection. It stands to reason that many groups worldwide have assayed the ability of inhibitors of protein translocation to prevent viral particle formation. Through an elegant influenza virus–host interactome analysis during infection, Heaton et al. proved for the first time in 2016 that Sec61 is one of the most effective interactors of several influenza virus proteins. The group’s analysis was not limited to influenza, and by pharmacologic inhibition and genetic knockdown of Sec61 they showed that an active translocon is required for the replication of influenza, HIV and dengue viruses [43]. Subsequently, our laboratory used the influenza virus model to prove that mycolactone can target virus envelope proteins just as well as endogenous targets, with specificity for type I/II but not type III TMPs [21]. Expanding the scope of its anti-viral activity, mycolactone was employed by Monel et al. to help understand the mechanisms of the cytopathic effects of the Zika virus. In this context, it was revealed that Sec61 activity drives the Zika virus-induced cell death program. Translocon inhibition through mycolactone prevented viral replication, the formation of the characteristic ER-derived vacuoles, and the ensuing cell death [44]. This study highlighted novel host–virus interaction mechanisms and possible pharmaceutical targets to prevent Zika virus-induced cytopathy, which can cause serious problems when the infection occurs during pregnancy, in some cases leading to birth defects and even fetal lethality. Along this same line, following a sequential affinity purification-mass spectrometry (AP-MS) analysis and RNAi screening aimed at generating a dengue–human protein–protein interaction map, Shah et al. revealed that the translocon machinery is a key interactor of dengue and Zika virus transmembrane proteins. Interestingly, Sec61 inhibition through cotransins not only completely abolished viral protein production in mammalian cells, but also inhibited dengue and Zika viral particle production and replication in cells derived from mosquitoes, the transmission vectors of these viruses [45]. These results identified Sec61 as a targetable host factor against dengue and Zika infections in two different hosts, humans and mosquitoes, amplifying their potential and interest.

Sec61 blockers even came under the spotlight during the recent COVID-19 pandemic. Driven again by a screen aimed at identifying protein–protein interactions between SARS-CoV-2 and human proteins, Sec61 was identified as one of the leading druggable human protein candidates to prevent such interactions. Multiple SARS-CoV-2 proteins were predicted to access the ER through Sec61 and in fact, by chemically blocking the translocon activity, the viral replication was inhibited in a virus plaque assay [46]. Ipomoeassin-F is a synthetic derivative of a glycoresin isolated from the leaves of Ipomoea squamosa [47], subsequently found to be another potent Sec61 inhibitor [48]. O’Keefe et al. [49] recently demonstrated through in vitro assays that ipomoeassin-F is a potent anti-SARS-CoV-2 agent. As a matter of fact, through the blockade of Sec61, ipomoeassin-F inhibited the expression of the viral proteins spike and ORF8 while at the same time inducing the degradation of the angiotensin-converting enzyme 2, a key host cell receptor that allows SARS-CoV-2 viral entry [49]. Therefore, Sec61 inhibitors show a dual action by decreasing both SARS-CoV-2 virion production and the cell entry receptor, promising efficacity against the early and late stages of viral infection. The discovery of novel anti-viral drugs with new modes of action is essential, given the emergence of drug resistance in existing viral infections and the threat of future pandemics. With regard to this, Sec61 inhibitors showed broad spectrum anti-viral activities, by acting both on the formation of essential viral proteins and by inhibiting viral entry in the host through different mechanisms. We believe that Sec61 blockers have the potential to be novel and powerful anti-viral tools.

## 8. Conclusions and Perspectives

Mycolactone may have started its journey through research as a detrimental mycobacterial toxin, but it subsequently turned out to be a powerful tool: not only has it provided us with many important lessons on Sec61 biology, but it is also paving the way for clinical applications of translocon inhibitors (Figure 1). Comparisons between mycolactone and other, less potent natural inhibitors of Sec61 such as cotransins suggest that it may be possible to achieve selective inhibition of protein targets. This may be used against clinically relevant Sec61 clients while bypassing the cytotoxicity issues of broad inhibitors. Importantly, natural inhibitors also provided structural information on the inactive state of Sec61 [22], which will be key in the development of small-molecule blockers of the translocon activity.

Mycolactone and the other natural inhibitors of Sec61 may not be suitable for employment in clinics due to their complexity, laborious synthesis and toxicity, but their activity and that of their derivatives have captured the attention of many researchers worldwide, who are already working on overcoming these issues. The race for the development of drug-like Sec61 inhibitors has already started [50,51] and, to date, Oncology is the primary indication for the usage of these small molecules, which have the potential to be added to the treatment portfolio of many cancer types to target multiple tumor drivers at once. Furthermore, Sec61 blockers proved to possess potent anti-inflammatory activities by targeting the expression of several cytokines, chemokines and cytokines receptors, paving the way for the development of novel treatments for inflammatory pain and inflammatory diseases. In Virology, the development of translocon blockers to decrease viral titers is particularly relevant because Sec61 is a host factor, and that can limit the likelihood of viral resistance. However, the potential of Sec61 inhibitors as antivirals will have to be confirmed in animal models of infection.

## Data Availability

No new data were created or analyzed in this study.
